# Can Getting Enough Vitamin D during Pregnancy Reduce the Risk of Getting Asthma in Childhood?

**DOI:** 10.3389/fped.2017.00087

**Published:** 2017-04-26

**Authors:** Evangelia Bountouvi, Konstantinos Douros, Anna Papadopoulou

**Affiliations:** ^1^Third Department of Pediatrics, Athens University Medical School, University General Hospital “Attikon”, Athens, Greece

**Keywords:** vitamin D, asthma, childhood, pregnancy outcome, vitamin D supplementation

## Abstract

The worldwide increase in asthma prevalence during the last decades and the re-emergence of vitamin D deficiency in many populations hinted toward an underlying association between these two conditions. Since asthma is presented with high incidence in childhood and neonatal vitamin D stores depend on maternal vitamin levels, a possible programming effect of maternal vitamin D status during gestation was suggested. Observational and longitudinal studies on this subject led to inconclusive results with glimmer of positivity. In the randomized controlled clinical trials (RCTs) that followed, increased doses of vitamin D were tested in pregnant women being at high risk of having an asthmatic child. Although, the results of RCTs showed a potential association with asthma-related phenotypes rather than asthma *per se*, the low toxicity of vitamin D supplements make it tempting to speculate that pregnant women at a high risk of obtaining a child with asthma may be benefited, especially if they are vitamin D deficient.

## Introduction

Epidemiological and experimental data on health impact of “multitasking” vitamin D support a protective effect of vitamin D over diseases such as cancer, cardiovascular, autoimmune, dementia, and diabetes (1, 2). Recently, randomized clinical trials have been conducted to determine optimal serum concentrations and optimal intake of vitamin D that might be beneficial to human health (3–5). The prevention of asthma development is one of the many potential health benefits of vitamin D (6). Since asthma is presented with high incidence in childhood, a possible programming effect of maternal vitamin D status during gestation has been proposed. Besides, pregnant women and infants are in high risk of vitamin D insufficiency (7–11). Several observational studies explored this issue and suggested a putative protective role of prenatal vitamin D sufficiency in the development of wheezing and asthma in the offspring [reviewed in Ref. (12)]. However, it remains unclear whether maternal vitamin D deficiency may contribute to the development of childhood asthma or it is the consequence of a generally altered biochemistry. In this mini-review article, we aim to present the current knowledge as it emerges from studies focused on the impact of maternal vitamin D status and vitamin D supplementation on the development of asthma in early childhood.

## Vitamin D Levels During Pregnancy

The main role of vitamin D during pregnancy is to maintain serum calcium levels by promoting calcium absorption in the intestine and allowing proper function of parathyroid hormone. It is, however, interesting that, during this period, vitamin D metabolism is shifted from the classical determinants of calcium homeostasis to other regulatory mechanisms. Although 25(OH)D circulating levels do not substantially differentiate or may be reduced compared to non-pregnant women, serum concentrations of vitamin D active metabolite, 1,25(OH)_2_D, are significantly elevated from early pregnancy (13, 14). This increased production possibly depends on 25(OH)D substrate availability (2, 15). It should be noticed that high maternal intake of 25(OH)D or exceeding vitamin D serum levels during pregnancy is not complicated from either hypercalcemia or hypercalciuria (16). Moreover, 1,25(OH)_2_D synthesis by 1-α-hydroxylase (CYP27B1) may be triggered by other modifiers, which are increased during gestation, such as parathyroid hormone-related protein, prolactin, estradiol (17), calcitonin (18–20), and placental lactogen. Furthermore, an autocrine or paracrine placental function of upregulated CYP27B1 (21–24) and methylation of CYP24A1 gene, which induces lower levels of catabolic 24-hydroxylase, have also been suggested to contribute to the exceeding levels of 1,25(OH)_2_D in pregnancy. Circulated 25(OH)D concentration of 40 ng/ml (100 nmol/l), more likely to be achieved by 4,000 IU D3 daily intake, has been proposed as the cutoff level to optimize maternal 1,25(OH)_2_D levels (15).

1,25(OH)_2_D does not pass through the hemochorial placenta, and fetal levels of 1,25(OH)_2_D are expected to be lower than the maternal (17). Cord blood levels of 25(OH)D correlate with 1,25(OH)_2_D whereas this is not the case for maternal circulation (25). Contrary to 1,25(OH)_2_D, 25(OH)D and its catabolic product, 24,25(OH)_2_D, readily cross the placenta. Consequently, the infant basically depends on mother’s 25(OH)D stores. Indeed, a strong correlation between maternal and cord blood 25(OH)D concentrations has been highlighted (26, 27). As a result, vitamin D deficiency during pregnancy exerts significant impact on vitamin D status of newborns and subsequently on early childhood (9, 28).

## The Impact of Vitamin D Exposure *In Utero*

The dependence of the developing fetus on maternal vitamin D status together with the reported extra skeletal function of the hormone and vitamin D deficiency epidemic during the last years led to further investigation of the consequences of maternal vitamin D insufficiency on pregnancy outcomes (6, 29, 30). Gestational vitamin D has now been linked to a variety of diseases such as osteoporosis, autoimmune disorders, altered brain development and adult mental health, food allergies, and asthma (17, 29, 31–33). The most cited adverse outcome of maternal vitamin D deficiency is neonatal rickets, while mild hypovitaminosis D could be more pronounced in exclusively breast-fed infants, on the basis that breast milk constitutes a poor source of vitamin D compared with formulas (34–36). In the light of the possible parallel-asthma epidemic, cellular molecular studies along with epidemiological data suggest that vitamin D deficiency may have a programming effect *in utero* and promote a trajectory to develop asthma in childhood and later in life (2, 37–39). An immunodulatory role of vitamin D has also been described in both immune system and lung cell maturation, which occurs mainly during fetal and early extrauterine life (40–42).

## Maternal Vitamin D Status and Associations with Asthma in Early Life

The putative effect of prenatal exposure to vitamin D on asthmatic phenotypes in early life has been initially investigated *via* observational studies based on either maternal dietary intake of vitamin D or 25(OH)D levels in venous maternal and/or cord blood [reviewed in Ref. (12)]. Mainly, the food-approached studies have suggested a protective role of vitamin D intake on the development of wheezing and asthma in offspring, with an attenuated effect after adjustment for multiple covariables (43–48). Despite weak associations, meta-analyses and systemic reviews replicated the potential link, warranting further investigation (49–52). Consequently, longitudinal studies measuring 25(OH)D levels, a possible more objective indicator of vitamin D status, were designed to explore the possible impact of vitamin D on asthma-like symptoms or asthma. However, these derived data failed to consistently replicate this inverse connection. A spectrum of all possible associations has accrued, demonstrating no association (53–57), an inverse (58–61), a *U*-curved (62, 63), and even a direct relationship (64, 65) between the two entities. In accordance, systematic reviews and meta-analyses on these birth cohort studies reproduced inconclusive evidence, revealing no association with asthma or wheezing (66), a *U*-shaped association with a lower risk of childhood asthma at maternal 25(OH)D levels of 30 ng/ml (70 nmol/l) (67) and a borderline significant inverse relationship (68).

Inconsistencies across studies have been attributed to a plethora of factors such as diverse study designs, missing significant follow-up, lack of a clear definition of vitamin D sufficiency in pregnancy, remarkable fluctuations in populations in different latitudes, genetic heterogeneity, the diversity of assays employed (69), low repeatability of serum 25(OH)D values (37), variety of vitamin D status determinants, multifactorial nature and phenotypical diversity of asthma, as well as imprecision in diagnosing asthma in early life. Moreover, the majority of the cohorts assessed a single measurement of 25(OH)D in cord blood or late pregnancy, which might not sufficiently reflect the virtual vitamin status during gestational dynamic process. Of note, food base approaches may be assumed as a better representative of long-term vitamin D intake, which might influence the fetus during all stages of gestation and even *ex utero*.

The inconsistent body of evidence with glimmer of positivity from observational prospective cohorts, the rather low toxicity of vitamin D, which is a relative inexpensive candidate in the attenuation of the asthma’s epidemic burden on public health, were clearly indicative of the need to proceed to clinical trials. Moreover, both animal and *in vitro* experiments have demonstrated a positive impact of prenatal vitamin D exposure on aspects of lung function and the process of inflammation, which are considered to contribute to asthma pathogenesis (70–72).

The first interventional study was conducted by Goldring et al. in UK (Table 1). The sample consisted of women with various ethnicities. Daily vitamin consumption of 800 IU D2 during the last trimester of pregnancy did not reveal any association with lung function, airway inflammation, and recurrent or asthma-predicting wheezing at 3 years of age, in comparison with the control group (73). In a recent randomized placebo-controlled double-blind trial in New Zealand, vitamin D was administrated in unselected pregnant women from the 27 weeks of gestation and subsequently in their infants during the first 6 months of life (placebo/placebo, 1,000/400 IU, and 2,000/800 IU). Despite that there was no difference preserved in the offspring’s 25(OH)D level at the age of 18months, a statistically significant lower proportion of children, belonged to vitamins D groups, were sensitized to mite antigens, as indicated by specific IgE measurements. Besides, a possible effect of vitamin D was depicted on primary care visits, where asthma was diagnosed by physicians (74). However, these clinical trials were biased by many limitations, such as small size, vitamin D administration only in the last trimester of pregnancy, possibly inadequate maternal dose administration, while authors also acknowledged that they were not primary designed to assess the specific outcome. Moreover, the first trial was not double blind and pertaining to the second one, a diagnosis of asthma at the age of 18 months was rather vague. Still, the association of vitamin D with asthma, ambiguously emerged from observational studies, could not be confirmed and established as a causative factor. Thus, further well-designed, randomized, controlled double blind trials followed to overcome the limitations of the previous studies and address the issue.

**Table 1:** **Randomized control trials on vitamin D supplementation during pregnancy and asthma/wheezing**.

Reference	Country and enrollment period	Study population	Intervention arms	Number/age of children at outcome assessment	Intake of intervention from/until	Effect on median vitamin D levels (ng/ml)	Outcomes of interest	Main findings
Goldring et al. (73)	UK 2007	180 mother–child pairs 4 Ethnic groups: Asian, Middle Eastern, Black and White	*Not blinded* Women: no treatment (*N* = 60) 800 IU (*N* = 60) ergocalciferol daily 200,000 IU (*N* = 60) cholecalciferol single bolus oral dose	158/3 years	Mothers: 27 weeks of gestation to delivery	Higher in cord blood of supplementary arm (control 6.8, daily dose 10.4, single dose 10 *p* < 0.001)	*Blinded* Wheezing, eczema, food allergy, rhinitis, atopy, URTI, LRTI, inhaled bronchodilator or steroids	No significant difference between groups in risk of wheezing at 3 years of age (RR: 0.86, 95% CI 0.49–1.50 *p* = 0.69) No significant difference between groups pertaining to rest secondary outcomes
Grant et al. (74)	New Zealand 2010–2011	260 mother–child pairs	*Double-blinded* Women/infant: placebo/placebo 1,000/400 IU 2,000/800 IU	185/18 months	Mothers: 27 weeks of gestation to delivery Off springs: birth to 6 months	*Enrollment*: 25 → no difference (*p* = 0.19) *Cord blood*: 27 vs 15 → higher in both supplemented groups than in placebo *6 months of age*: 40 vs 30 → higher vit D group had the highest and placebo group the lowest concentrations *18 months of age*: 24.4 → no difference (*p* = 0.30)	Secondary outcomes Sensitization to airborne allergens Primary health care visits for respiratory illnesses	Decreased proportion of children sensitized to four mites antigens Study group differences in the proportion of children with primary care visits of doctor-diagnosed asthma (11, 0, and 4%, *p* = 0.002)
Chawes et al. (75)	Denmark 2009–2010	623 mother–child pairs recruited from COPSAC_2010_ cohort study	*Double-blinded* Placebo + 400 IU (*N* = 308) Cholecalciferol 2,400 + 400 IU (*N* = 315)	581/3 years	Mothers: 24 weeks of gestation to 1 week after delivery	Increase in maternal serum vitD level in treatment group (mean SD at randomization vs postpartum: 31 vs 43; control group 31 vs 29) Mean group difference 13 [95% CI 11–16, *p* < 0.001]	Persistent wheeze, asthma, URTI, LRTI, episodes of lung symptoms, SPT, specific IgE	No significant reduction in the risk of persistent wheezing per 4 ng/ml increase in maternal serum vitamin D level (HR: 0.86, 95% CI 0.89–0.99 *p* = 0.03) Reduced number of troublesome lung episodes (IRR: 0.83, 95% CI 0.71–0.97 *p* = 0.02) Upregulation or airway immune profile (principal component analysis *p* = 0.04) No effect on additional end points
Litonjua et al. (76) (VDAART)	USA 2009–2011	876 mothers at high risk of having child with asthma	*Double-blinded* Placebo + 400 IU (*N* = 436) 4,000 + 400 IU (*N* = 440)	748/3 years	Mothers: between 10 and 18 weeks of gestation to delivery	*Third trimester*: higher in supplemented group vs control (39.2 vs 26.8, mean difference 12.4; 95% CI 10.5–14.3, *p* < 0.001) *Cord blood*: significant difference preserved *1 and 3 years postpartum*: no difference	Wheezing or asthma, eczema with rash, LRTI, mean total IgE. Aeroallergens sensitization, specific IgE	No significant reduction in the incidence of asthma and recurrent wheezing by 6.1% (HR: 0.8, 95% CI 0.6–1.0 *p* = 0.51)

*CI, confidence interval; HR, hazard ratio; RR, relative risk; SPT, skin prick test; RI, respiratory infection; URTI, upper respiratory tract infection; LRTI, lower respiratory tract infection*.

Recently, the results of two randomized controlled clinical trials (RCTs) were presented. In Denmark, Chawes et al. recruited 623 unselected women who were randomized to receive either 2,800 or 400 IU (as part of routine care) of vitamin D daily from 24 weeks of gestation until the first postpartum week. Adherence was assessed by returned pills and the follow-up rate was 94%. The primary outcome was persistent wheezing in the offspring in the first 3 years of age. A no significant 4% absolute reduction in incidence of wheezing was reported in the children whose mothers were assigned to 2,800 IU group (hazard ratio, 0.76 [95% CI 0.52–1.12]). Pertaining to secondary outcomes, the number of troublesome lung symptoms was significant lower in children of the intervention arm. However, no association was observed with asthma or other allergic diseases at 3 years of age (75). The second multicenter double-blind RCT was conducted in USA by Litonjua et al. Pregnant women on high risk to obtain children with asthma were randomized to either an interventional arm receiving 4,400 IU of vitamin D or to a control arm receiving 400 IU, as usual care, from 10 to 18 weeks of gestation until delivery. Compliance was measured by electronic medication containing caps. Ultimately, 748 children from the 876 initially enrolled mothers were followed up to 3 years of age. The cumulative incidence of asthma or wheezing was 6.1 lower in the intervention group in the first 3 years of life, but this was a borderline result, which did not meet statistical significance [hazard ratio, 0.8 (95% CI 0.6–1.0)]. Moreover, this effect was reported to be attenuated with increasing age. Of note, despite the intervention significantly increased circulating vitamin D levels in women, 1-year postpartum no difference was preserved (76).

Although the results of both RCT did not meet statistical significance, the wide confidence intervals, which include a clinically important protective effect suggest that the studies may have been underpowered to measure the primary outcomes. A recent meta-analysis, which processed these data from both these two RCT and the study of Goldring et al., suggested that prenatal daily supplement of vitamin D might have a positive effect on childhood wheezing [relative risk, 0.812 (95% CI 0.673–0.98)] (77). Given the fact that wheezing, usual viral-induced, in early childhood is a frequent cause of hospitalization and morbidity (78), further studies to confirm the possible causality are warranted.

Taken all together, vitamin D shows a potential association rather with asthma-related phenotypes than with asthma *per se*. For instance, in early childhood, wheezing is frequent transient or viral induced and 60% of these children are not expected to develop asthma (79). Therefore, it remains to be answered if a real cause-effect relationship exists or vitamin D exerts a protective role *via* other underlying mechanisms of this multifactorial disease, such as lung function or protection against viral infections. Additionally, vitamin D is a marker of UV exposure, which can modulate immunity *via* non-vitamin D related pathways (80, 81). Furthermore, as far as asthma diagnosis is ambiguous at the first 3 years of life, long-term follow-up may contribute to explore the possible relationship. However, the attenuation of the observed effect with aging (59, 74, 76) complicates this theory. Notably, a large prospective observational birth cohort with long-term follow-up until early adulthood did not reveal a protective programming effect of high maternal 25(OH)D concentration in the third trimester of pregnancy on asthma at the age of 20 years, whereas maternal 25(OH)D levels over 50 ng/ml (125 nmol/l) were associated with higher risk of asthma hospitalization during the first 25 years of life (65). Moreover, it has been proposed that studies should focus on vitamin D status in early pregnancy and supplementary administration should be initiated from the first trimester of gestation in order to observe the optimal effect on risk of childhood asthma, though evidence from Chawes et al.’s clinical trial did not confirm this notion (75). In addition, an interesting remark is that study designs and analyses do not take into consideration the starting maternal vitamin D status and dose adequacy for the different body systems. This is also an issue that should be addressed.

Irrefutably, both entities of asthma and vitamin D deficiency are multifactorial. A plethora of environmental and genetic/epigenetic factors contribute in a complex manner to a partly understood pathogenesis, while the shifted vitamin D metabolism during pregnancy is also not fully elucidated. As a result, unmeasured aspects and residual confounding factors are rather difficult to be diminished from the studies. Besides, even if the putative effect suggested by the current RCTs be strengthened by further investigations, this would only explain a little proportion of asthma epidemic. Shifting the discussion to a clinical platform, vitamin D administration during pregnancy has not yet been justified. However, given the rather low toxicity of such a supplement and based on the evidence derived of the two RCTs, pregnant women at a high risk of obtaining a child with asthma may be benefited, particularly if they are deficient (82). Moreover, any intervention should be individualized to initial maternal vitamin D status and be followed up, since a *U*-curve association between vitamin D circulating levels and impact on allergy and human health has been also proposed (83, 84).

## Key Elements

- Vitamin D shows a potential association rather with asthma-related phenotypes than with asthma *per se*. Further well-designed RCTs to confirm the possible associations are warranted.
- Vitamin D supplementation during pregnancy has not yet been justified. However, given the rather low toxicity of such a supplement, pregnant women at a high risk of obtaining a child with asthma may be benefited, especially if they are vitamin D deficient. Any intervention should be individualized to initial maternal vitamin D status and be followed up.

## Author Contributions

AP contributed to the conception of the work, the revision, and the final approval of the manuscript. EB contributed to the design of the work, the drafting, and the final approval of text manuscript. KD contributed to the design of the work, the revision, and the final approval of the manuscript. All the authors agreed to be accountable for all aspects of the work.

## Conflict of Interest Statement

The authors declare that the research was conducted in the absence of any commercial or financial relationships that could be construed as a potential conflict of interest.
